# Angiopoietin-like protein 3 and 4 in obesity, type 2 diabetes mellitus, and malnutrition: the effect of weight reduction and realimentation

**DOI:** 10.1038/s41387-018-0032-2

**Published:** 2018-04-25

**Authors:** Anna Cinkajzlová, Miloš Mráz, Zdeňka Lacinová, Jana Kloučková, Petra Kaválková, Helena Kratochvílová, Pavel Trachta, Jarmila Křížová, Denisa Haluzíková, Jan Škrha, Hana Papežová, Martin Haluzík

**Affiliations:** 1 Centre for Experimental Medicine, Institute for Clinical and Experimental Medicine, Prague, Czech Republic; 2 Department of Medical Biochemistry and Laboratory Diagnostics, First Faculty of Medicine and General University Hospital, Charles University in Prague, Prague, Czech Republic; 3 Diabetes Centre, Institute for Clinical and Experimental Medicine, Prague, Czech Republic; 4 Third Department of Medicine—Department of Endocrinology and Metabolism, First Faculty of Medicine and General University Hospital, Charles University in Prague, Prague, Czech Republic; 5 Department of Sports Medicine, First Faculty of Medicine and General University Hospital, Charles University in Prague, Prague, Czech Republic; 6 Department of Psychiatry, First Faculty of Medicine and General University Hospital, Charles University in Prague, Prague, Czech Republic

## Abstract

**Background:**

Angiopoietin-like proteins (ANGPTLs) 3 and 4 are circulating factors that participate in the regulation of lipid and glucose metabolism.

**Subjects and methods:**

We measured serum ANGPTL3 and 4 levels in 23 patients with obesity, 40 patients with obesity and type 2 diabetes mellitus (T2DM), 22 patients with anorexia nervosa (AN), 15 subjects undergoing 72-h fasting, and 12 patients with short bowel syndrome (SBS), and their changes after very-low-calorie diet (VLCD), bariatric surgery, partial realimentation, acute fasting, and parenteral nutrition in order to assess their possible role in metabolic regulations.

**Results:**

Serum ANGPTL4 levels were higher in obese subjects without/with T2DM (94.50 ± 9.51 and 134.19 ± 7.69 vs. 50.34 ± 4.22 ng/ml, *p* < 0.001) and lower in subjects with AN relative to healthy control subjects (38.22 ± 4.48 vs. 65.80 ± 7.98 ng/ml, *p* = 0.002), while serum ANGPTL3 levels demonstrated inverse tendency. Nutritional status had no effect on ANGPTL3 and 4 mRNA expression in adipose tissue. Fasting decreased ANGPTL3 and increased ANGPTL4 levels, while VLCD reduced only ANGPTL3. Bariatric surgery and realimentation of AN or SBS patients had no effect on either ANGPTL. Multiple regression analysis identified BMI as an independent predictor of ANGPTL3; and BMI and HbA_1c_ as independent predictors of ANGPTL4, respectively.

**Conclusions:**

Taken together, our data suggest that serum ANGPTL3 and 4 levels are influenced by nutritional status and fasting and could be involved in the metabolic disturbances present in obesity and AN.

## Introduction

Angiopoietin-like proteins (ANGPTLs) 3 and 4 are members of the ANGPTL protein family named according to their structural similarity with angiopoietins^1^. However, ANGPTLs do not bind to angiopoietin-specific receptors^2^ and are thus still considered orphan ligands. Both ANGPTL4 (peroxisome proliferator-activated-γ angiopoietin-related protein or fasting-induced adipose factor) and ANGPTL3 have been shown to affect a number of biological processes, including angiogenesis^3,4^, hematopoietic stem cell activity^5,6^, cancer cell invasion^7,8^ as well as regulation of lipid and glucose metabolism^9,10^.

ANGPTL3 and 4 are released into systemic circulation primarily from the liver and act in a number of tissues, including white adipose tissue (WAT)^2^. In spite of their similar structure with 31% amino-acid sequence identity^11^, their biological actions differ according to underlying conditions^12–14^. ANGPTL3 is upregulated by liver X receptor, which serves as a sensor of cholesterol metabolism and lipid biosynthesis^15,16^, while ANGPTL4 is a downstream target of peroxisome proliferator-activated receptors that modulate lipid metabolism, insulin sensitivity, and adipocyte differentiation. Both ANGPTL3 and ANGPTL4 also inhibit lipoprotein lipase (LPL) and stimulate lipolysis^17^.

In spite of these data, the exact roles of ANGPTL3 and 4 in glucose and lipid metabolism in humans are still only partially understood. In our study, we therefore explored the changes of ANGPTL3 and ANGPTL4 circulating levels and their mRNA expression in subcutaneous adipose tissue (SAT) of obese subjects with and without type 2 diabetes mellitus (T2DM) as well as chronically malnourished individuals with anorexia nervosa (AN) or short bowel syndrome (SBS) as models of severe malnutrition of different etiology. We further evaluated the effects of selected energy balance-modifying interventions, including acute fasting, very-low-calorie diet (VLCD), bariatric surgery, and partial enteral and parenteral realimentation on both ANGPTLs in order to evaluate their potential role in metabolic improvements associated with these procedures and gain more insight into their suggested metabolic regulatory functions.

## Materials and methods

### Study subjects and interventions

Twenty-three patients with simple obesity (OB group), 40 obese individuals with T2DM (T2DM group, 27 of which underwent VLCD and 13 bariatric surgery), 22 patients with AN (AN group), 15 subjects undergoing a 72-h fast to rule out organic hyperinsulinism, and 12 subjects with SBS (SBS group) were enrolled into the study. Two age-matched healthy lean control groups (1 with 22 subjects for OB and T2DM individuals and a second one with 15 subjects for AN group) were included as well. Written informed consent was obtained from each subject prior to enrollment. The study was approved by the Human Ethics Review Board, First Faculty of Medicine and General University Hospital, Prague, Czech Republic.

### Anthropometric examination, blood, and adipose tissue sampling

All subject included in the study were measured and weighted, and their body mass index (BMI) was calculated. Blood samples for biochemical and hormonal measurements were taken after overnight fasting and were centrifuged for 10 min at 1000 × *g* within 30 min from collection. Aliquots were stored at −80 °C.

Samples of SAT were obtained by needle aspiration biopsy from abdominal region or from laparoscopic channel or laparotomy in case of surgery after overnight fasting. In subjects undergoing bariatric surgery visceral adipose tissue (VAT) samples were taken from abdominal cavity at the beginning of the procedure. Samples were subsequently stored at −80 °C.

### Very-low-calorie diet

Twenty-three obese patients and 27 obese patients with T2DM underwent a 3-week VLCD period. The VLCD diet consisted of 50 g of carbohydrates (15–20 g in mono- and disaccharides, and 30–35 g in polysaccharides), 20 g of lipids (6–8 g unsaturated and 12–14 g saturated), 55 g of proteins, and 20 g of fiber accounting for a total energy content of 2500 kJ/day. Anthropometric measurements and blood and SAT samples were taken before and after VLCD.

### Bariatric surgery

Thirteen obese patients with T2DM underwent bariatric surgery. The procedures included gastric plication (10 subjects), gastric banding (2 subjects), and gastric bypass (1 subject). Anthropometric measurements and blood and SAT samples were taken at baseline (visit 1) and 1 (visit 2), 6 (visit 3), and 12 months after surgery (visit 4). In addition to SAT obtained during each visit, samples of SAT and VAT were taken during surgery for the evaluation of depot-specific differences.

### Partial realimentation in subjects with AN

Twenty-two patients with AN were included into this substudy. The diagnosis of eating disorder was based on Diagnostic Statistical Manual IV. The mean duration of AN was 9.0 ± 1.8 years. Subjects were free of any medication for more than 3 months prior to the beginning of the study. The realimentation program has been described in more detail previously^18^. During the realimentation period (46 ± 2 days) the daily energy content was 9630 kJ/day. Anthropometric and blood samples were taken at baseline and after realimentation.

### Fasting in subjects with functional hypoglycemia

Fifteen non-obese non-diabetic subjects with suspected hypoglycemic episodes underwent a supervised 72-h fasting test to rule out organic hyperinsulinism, all of them with negative results. ANGPTLs were measured at the beginning (V1) and end of fasting (i.e., after 72 h—V2) and 2 h after realimentation (V3).

### Parenteral nutrition in SBS subjects

Twelve patients with SBS on parenteral nutrition were included into this substudy. Blood samples were taken at baseline (V1) and 14 days (V2), 6 (V3), and 12 months (V4) after hospitalization.

### Hormonal and biochemical assays

Serum ANGPTL3 and ANGPTL4 levels were measured by sandwich enzyme immunoassays using commercial ELISA kits (BioVendor, Brno, Czech Republic, Brno). The whole assays, including serum dilution were done according to the manufacturer’s protocol. Sensitivity was 1.08 ng/ml for ANGPTL3 and 0.173 ng/ml for ANGPTL4. Serum leptin concentrations were measured by commercial ELISA kit (Biovendor). Sensitivity was 0.2 ng/ml. Serum high-sensitivity C-reactive protein (hsCRP) levels were measured by high-sensitive ELISA (Bender Medsystems, Vienna, Austria) with sensitivity of 3 pg/ml. Insulin concentrations were measured by RIA kit (Cis Bio International, Gif-sur-Yvette, France). Sensitivity was 2.0 µIU/ml. The intra- and interassay variabilities were <5.0 and 10.0%, respectively.

Biochemical parameters (blood glucose, glycated hemoglobin—HbA_1c_, total and high-density lipoprotein (HDL) cholesterol, triglycerides, and CRP) were measured and low-density lipoprotein (LDL) cholesterol was calculated by standard laboratory methods. The homeostasis model assessment (HOMA) was calculated according to equation: (fasting serum insulin (mIU/l) × fasting serum glucose (mmol/l))/22.5.

### Quantitative real-time PCR

SAT and VAT samples were homogenized on MagNA Lyser Instrument (Roche Diagnostics GmbH, Mannheim, Germany). Total RNA from homogenized tissue was extracted on MagNA Pure instrument using MagNa Pure Compact RNA Isolation kit (tissue; Roche Diagnostics GmbH). RNA concentration was determined from absorbance at 260 nm on a NanoPhotometer (Implen, Munchen, Germany). Reverse transcription was performed using random primers according the manufacturer’s protocol of the High-Capacity cDNA Reverse Transcription Kits (Applied Biosystems, Foster City, CA, USA). The input amount of RNA was 750 μg/reaction. Gene expression was performed on a 7500 Real-Time PCR System (Applied Biosystems). For reaction a mix of TaqMan^®^ Universal PCR Master Mix II, NO AmpErase^®^ UNG (Applied Biosystems), nuclease-free water (Fermentas Life Science, Vilnius, Lithuania), and specific TaqMan^®^ gene Expression Assays (Applied Biosystems) was used. Beta-2 microglobulin was applied as endogenous reference. The formula 2^−ddCt^ was used to calculate relative gene expression. For determination of ANGPTL4 mRNA expression diluted samples were used, while for ANGPTL3 mRNA expression samples remained undiluted.

### Statistical analysis

Statistical analysis was performed on SigmaStat 3.0 and SigmaPlot 8.0 software (SPSS Inc., Chicago, IL, USA). Anthropometric, biochemical, and hormonal results are expressed as means ± standard error of the mean. One-way analysis of variance (ANOVA)/one-way repeated measures (RM) ANOVA followed by Holm-Sidak test, one-way ANOVA on ranks/one-way RM ANOVA on ranks followed by Dunn’s method, unpaired *t*-test or Mann–Whitney rank sum test, and paired-test or Wilcoxon signed-rank were used for the assessment of intergroup differences, as appropriate. Spearman or Pearson correlation test was used to calculate the relationships between serum ANGPTL3 and 4 levels or mRNA expression and other parameters. Combined groups of all study subjects with age-matched control subjects were used for correlation analysis. Statistical significance was assigned to *p* < 0.05. Multiple linear regression analysis using the backward stepwise variable selection method was performed using parameters with significant results from Spearman or Pearson correlation test.

## Results

### Very-low-calorie diet

At baseline, obese patients with and without T2DM had higher BMI, insulin, leptin, hsCRP, and HOMA index relative to control subjects, while T2DM patients also showed increased blood glucose, HbA_1c_, and triglycerides, and reduced HDL cholesterol (Table 1). Non-diabetic subjects had lower BMI, blood glucose, HbA_1c_, HOMA index, triglycerides, leptin, and hsCRP, and higher HDL cholesterol at baseline compared to diabetics enrolled in the VLCD substudy.

**Table 1:** Anthropometric, biochemical, and hormonal characteristics of patients with obesity and obesity/type 2 diabetes mellitus undergoing VLCD

	Controls	OB before	OB after	T2DM before	T2DM after
Number	22	23	23	27	27
Age (year)	51.6 ± 1.33	51.4 ± 2.10	NP	55.4 ± 1.54	NP
Body mass index (kg/m^2^)	23.4 ± 0.44	44.9 ± 2.15*	42.5 ± 2.15*^,^**	50.9 ± 1.63*^,^***	47.9 ± 1.52*^,^**^,^***
Blood glucose (mmol/l)	4.91 ± 0.08	5.39 ± 0.20	5.00 ± 0.11**	9.46 ± 0.80*^,^***	7.31 ± 0.50*^,^**^,^***
HbA_1c_ (mmol/mol)	37.5 ± 0.81	41.9 ± 1.22	NP	71.4 ± 4.67*^,^***	NP
Total cholesterol (mmol/l)	5.18 ± 0.22	5.00 ± 0.30	4.58 ± 0.27	4.85 ± 0.25	3.85 ± 0.16*^,^**^,^***
Triglycerides (mmol/l)	1.12 ± 0.12	1.61 ± 0.20	1.26 ± 0.14	2.26 ± 0.27*^,^***	1.85 ± 0.16*^,^***
HDL cholesterol (mmol/l)	1.65 ± 0.07	1.38 ± 0.08	1.31 ± 0.09*	1.08 ± 0.04*^,^***	1.03 ± 0.11*^,^**^,^***
LDL cholesterol (mmol/l)	3.03 ± 0.17	2.90 ± 0.22	2.70 ± 0.21	2.80 ± 0.20	2.05 ± 0.16*^,^**^,^***
Insulin (mUI/l)	16.6 ± 0.76	39.7 ± 4.73*	33.8 ± 2.70*	52.3 ± 9.23*	41.5 ± 3.94*
Leptin (ng/ml)	13.3 ± 2.00	47.3 ± 5.05*	40.8 ± 5.05*^,^**	61.3 ± 3.81*^,^***	53.7 ± 4.39*^,^**^,^***
hsC-reactive protein (mg/l)	0.20 ± 0.05	1.51 ± 0.40*	1.22 ± 0.34*^,^**	2.34 ± 0.28*^,^***	1.41 ± 0.21*^,^**
HOMA index	3.64 ± 0.20	9.93 ± 1.42*	7.67 ± 0.69*^,^**	20.82 ± 3.65*^,^***	11.83 ± 1.18*^,^**^,^***
ANGPTL4 mRNA expression in SAT	1.04 ± 0.09	1.23 ± 0.17	1.44 ± 0.16	1.07 ± 0.08	1.10 ± 0.13

Values are mean ± SEM

*OB* non-diabetic obese subjects, *T2DM* obese subjects with T2DM, *NP* non-measured parameter

*p < 0.05 vs. controls, one-way ANOVA/ANOVA on ranks; **p < 0.05 vs. before VLCD, paired *t*-test or Wilcoxon signed-rank test; ***p < 0.05 vs. non-diabetic obese subjects, unpaired *t*-test or Mann–Whitney rank sum test

VLCD reduced BMI, blood glucose, HOMA index, leptin, and hsCRP in both groups with additional decrease in total, HDL, and LDL cholesterol in T2DM subjects (Table 1).

### Bariatric surgery

At baseline, obese patients with T2DM enrolled in the bariatric surgery substudy had higher BMI, blood glucose, HbA_1c_, triglycerides, insulin, HOMA index, leptin, and hsCRP, and reduced HDL cholesterol relative to control subjects (Table 2).

**Table 2:** Anthropometric, biochemical, and hormonal characteristics of patients with obesity and type 2 diabetes mellitus undergoing bariatric surgery

	Controls	V1	V2	V3	V4
Number	22	13	13	13	13
Age (year)	51.6 ± 1.33	56.2 ± 2.13	NP	NP	NP
Body mass index (kg/m^2^)	23.4 ± 0.44	41.6 ± 1.69*	37.0 ± 1.63*	34.4 ± 1.79*^,^**	35.4 ± 1.79*^,^**
Blood glucose (mmol/l)	4.91 ± 0.08	10.2 ± 1.19*	6.47 ± 0.53*^,^**	6.69 ± 0.54*^,^**	8.12 ± 1.11*
HbA_1c_ (mmol/mol)	37.5 ± 0.81	66.3 ± 4.84*	54.6 ± 3.15*^,^**	53.4 ± 4.67*^,^**	53.9 ± 4.62*^,^**
Total cholesterol (mmol/l)	5.18 ± 0.22	5.35 ± 0.29	4.69 ± 0.35	4.96 ± 0.33	4.56 ± 0.16
Triglycerides (mmol/l)	1.12 ± 0.12	2.86 ± 0.73*	1.76 ± 0.24*^,^**	1.85 ± 0.28**	1.71 ± 0.26**
HDL cholesterol (mmol/l)	1.65 ± 0.07	1.21 ± 0.07*	1.09 ± 0.06*	1.26 ± 0.07*^,^***	1.29 ± 0.07*^,^***
LDL cholesterol (mmol/l)	3.03 ± 0.17	3.19 ± 0.33	2.81 ± 0.29	2.86 ± 0.26	2.50 ± 0.17
Insulin (mUI/l)	16.6 ± 0.76	97.1 ± 33.01*	65.7 ± 28.0*	72.5 ± 32.6*^,^**	64.0 ± 24.6*
Leptin (ng/ml)	13.3 ± 2.00	39.2 ± 5.82*	20.6 ± 4.10**	21.3 ± 5.18**	24.0 ± 5.71**
hsC-reactive protein (mg/l)	0.20 ± 0.05	3.88 ± 0.83*	2.04 ± 0.51*	2.50 ± 0.88*	1.61 ± 0.42*^,^**
HOMA index	3.64 ± 0.20	46.36 ± 19.67*	19.95 ± 8.93*^,^**	27.33 ± 14.26*^,^**	22.73 ± 9.36*
ANGPTL4 mRNA expression in SAT	1.04 ± 0.09	1.15 ± 0.17	1.25 ± 0.19	0.94 ± 0.15	1.10 ± 0.11

Values are mean ± SEM

*NP* non-measured parameter, *V1* before bariatric surgery, *V2* 1 month after bariatric surgery, *V3* 3 months after bariatric surgery, *V4* 1 year after bariatric surgery

*p < 0.05 vs. controls, one-way ANOVA/ANOVA on ranks; **p < 0.05 vs. V1, one-way repeated measures ANOVA/one-way repeated measures ANOVA on ranks; ***p < 0.05 vs. V2, one-way repeated measures ANOVA/one-way repeated measures ANOVA on ranks

Bariatric surgery significantly reduced BMI, HbA_1c_, triglycerides, leptin, and hsCRP levels, and these changes lasted until one year after the procedure (Table 2). A temporary improvement was also observed in fasting glucose, insulin, and HOMA index.

### Different types of malnutrition/negative energy balance—AN, SBS, and acute fasting

At baseline, patients with AN had lower BMI, blood glucose, insulin, and HOMA index compared with control subjects with a significant increase after realimentation (Table 3). Upon enrollment, subjects with SBS had normal BMI and glucose levels. Parenteral nutrition had no significant effect on any of the measured parameters, although a trend to increased BMI and reduced CRP was observed (Table 4). Patients undergoing acute fasting had normal baseline fasting glucose and lipid levels (data not shown).

**Table 3:** Anthropometric, biochemical, and hormonal characteristics of patients with anorexia nervosa: the effect of partial realimentation

	Controls	AN before	AN after
Number	15	22	22
Age (year)	22.9 ± 0.77	24.5 ± 1.24	NP
Body mass index (kg/m^2^)	21.9 ± 0.54	15.4 ± 0.26*	17.3 ± 0.24*^,^**
Blood glucose (mmol/l)	4.39 ± 0.08	4.02 ± 0.06*	4.28 ± 0.08**
Total cholesterol (mmol/l)	4.36 ± 0.20	7.66 ± 3.10	5.22 ± 0.26
Triglycerides (mmol/l)	0.98 ± 0.13	1.49 ± 0.14	1.44 ± 0.18
HDL cholesterol (mmol/l)	1.76 ± 0.10	1.59 ± 0.10	1.65 ± 0.10
LDL cholesterol (mmol/l)	2.18 ± 0.13	2.35 ± 0.17	2.93 ± 0.20*^,^**
Insulin (mUI/l)	19.3 ± 1.12	15.8 ± 0.79*	16.1 ± 1.03*^,^**
HOMA index	3.81 ± 0.27	2.78 ± 0.14*	3.12 ± 0.19*

Values are mean ± SEM

*AN* subjects with anorexia nervosa, *NP* non-measured parameter

*p < 0.05 vs. controls, one-way ANOVA/ANOVA on ranks; **p < 0.05 vs. before realimentation, paired *t*-test or Wilcoxon signed-rank test

**Table 4:** Anthropometric, biochemical, and hormonal characteristics of patients with short bowel syndrome

	SBS V1	SBS V2	SBS V3	SBS V4
Number	12	9	5	5
Age (year)	64.2 ± 4.3			
Body mass index (kg/m^2^)	24.09 ± 1.595	25.9 ± 2.32	25.1 ± 1.16	28.1 ± 3.01
Blood glucose (mmol/l)	5.133 ± 0.259	5.68 ± 0.28	5.75 ± 0.83	5.38 ± 0.35
HbA_1c_ (mmol/mol)	37.0 ± 1.874	N/A	N/A	33.2 ± 1.24
Total cholesterol (mmol/l)	3.577 ± 0.158	3.99 ± 0.37	2.83 ± 0.30	4.11 ± 0.27
Triglycerides (mmol/l)	1.881 ± 0.356	1.58 ± 0.36	1.64 ± 0.32	2.21 ± 0.54
HDL cholesterol (mmol/l)	0.922 ± 0.156	0.95 ± 0.17	0.89 ± 0.24	1.36 ± 0.35
LDL cholesterol (mmol/l)	1.894 ± 0.102	2.07 ± 0.17	1.78 ± 0.12	1.62 ± 0.32
Leptin (ng/ml)	9.499 ± 3.437	10.22 ± 3.66	9.87 ± 3.67	4.59 ± 0.72
C-reactive protein (mg/l)	72.717 ± 21.744	45.2 ± 20.2	43.2 ± 18.4	13.0 ± 4.70

Values are mean ± SEM

*SBS* subjects with short bowel syndrome, *N/A* non-applicable

### Effect of interventions on serum ANGPTL3 and 4 levels and adipose tissue mRNA expression

**Fig. 1:**
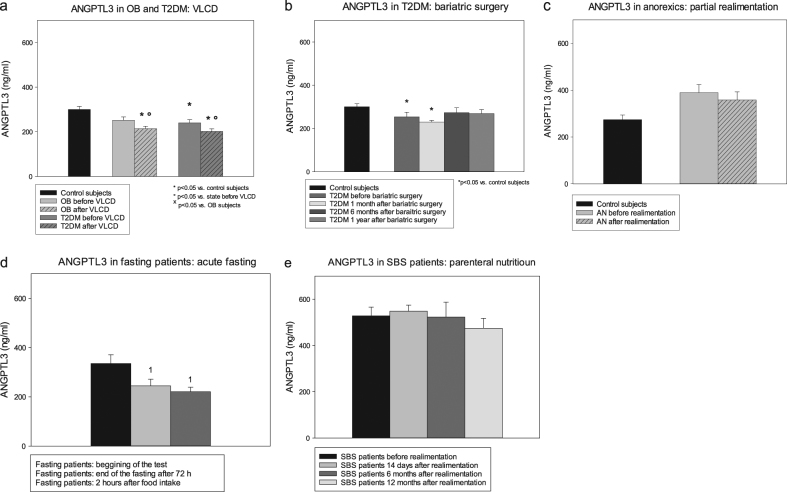
Serum angiopoietin-like protein 3 at baseline and after interventions. Changes of serum ANGPTL3 levels after very-low-calorie diet (**a**), bariatric surgery (**b**), partial realimentation (**c**), acute fasting (**d**), and parenteral nutrition (**e**). OB non-diabetic obese patients, T2DM obese patients with T2DM, AN patients with anorexia nervosa, SBS patients with short bowel syndrome. Values are mean ± SEM. *p < 0.05 vs. control subjects, one-way ANOVA/ANOVA on ranks; °*p* < 0.05 vs. before VLCD/realimentation, paired *t*-test or Wilcoxon signed-rank test; ^x^*p* < 0.05 vs. subjects with simple obesity, unpaired *t*-test or Mann–Whitney rank sum test; ¹*p* < 0.05 vs. V1, one-way RM ANOVA/one-way RM ANOVA on ranks

**Fig. 2:**
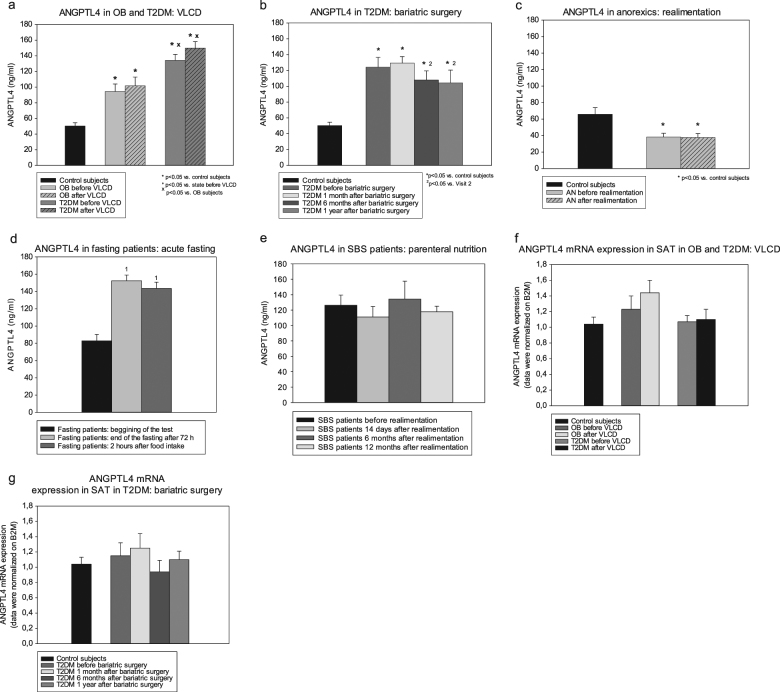
Serum angiopoietin-like protein 4 and its mRNA expression in subcutaneous adipose tissue at baseline and after interventions. Changes of ANGPTL4 levels after very-low-calorie diet (**a**), bariatric surgery (**b**), partial realimentation (**c**), acute fasting (**d**), parenteral nutrition (**e**), and changes of ANGPTL4 mRNA expression in SAT after very-low-calorie diet (**f**) and bariatric surgery (**g**). OB non-diabetic obese patients, T2DM obese patients with T2DM, AN patients with anorexia nervosa, SBS patients with short bowel syndrome. Values are mean ± SEM. *p < 0.05 vs. control subjects, one-way ANOVA/ANOVA on ranks; *p* < 0.05 vs. before VLCD/realimentation, paired *t*-test or Wilcoxon signed-rank test; ^x^*p* < 0.05 vs. subjects with simple obesity, unpaired *t*-test or Mann–Whitney rank sum test; ¹*p* < 0.05 vs. V1, one-way RM ANOVA/one-way RM ANOVA on ranks, ²*p* < 0.05 vs. V2, one-way RM ANOVA/one-way RM ANOVA on ranks

In the VLCD substudy, serum ANGPTL3 levels were at baseline lower in obese diabetic, but not in non-diabetic, patients compared with healthy controls (Fig. 1a), while ANGPTL4 was elevated in both obese subjects with and without T2DM (Fig. 2a). ANGPTL4 levels were also higher in T2DM relative to non-diabetic subjects. VLCD decreased ANGPTL3 levels (Fig. 1a), while having no effect on serum ANGPTL4 (Fig. 2a).

At baseline, obese diabetics enrolled in the bariatric surgery substudy had decreased ANGPTL3 (Fig. 1b) and increased ANGPTL4 levels (Fig. 2b) relative to control subjects. Bariatric surgery did not significantly affect ANGPTL3 levels (Fig. 2a), while reducing serum ANGPTL4 at months 6 and 12 relative to month 1 after procedure (Fig. 2b).

Before intervention, patients with AN showed lower ANGPTL4 and a trend toward higher ANGPTL3 levels relative to control subjects (Figs. 1c and 2c). Partial realimentation had no effect on serum ANGPTLs. Acute fasting decreased serum ANGPTL3 and simultaneously increased ANGPTL4 concentrations with subsequent food intake having no effect during the 2 h post fasting (Figs. 1d and 2d). Throughout the whole year parenteral nutrition did not influence either ANGPTL in SBS subjects (Figs. 1e and 2e).

ANGPTL4 mRNA expression in SAT was affected neither by the presence of obesity/T2DM nor by weight-reducing interventions (Fig. 2f, g). When compared between SAT and VAT no difference in ANGPTL4 mRNA expression was found in obese patients with T2DM undergoing bariatric surgery (*p* = 0.092). In contrast, ANGPTL3 mRNA expression was almost undetectable in adipose tissue (data not shown).

### Relationship of ANGPTLs to other parameters

In the combined group of all study subjects except SBS subjects, ANGPTL3 levels positively correlated with HDL cholesterol and inversely with BMI, blood glucose, triglycerides, insulin, HOMA index, and leptin (Supplemental Table 1). Multiple linear regression analysis revealed that BMI was the only independent predictor for ANGPTL3 levels (adjusted *R* = 0.153, *p* < 0.001).

ANGPTL4 levels positively correlated with BMI, blood glucose, HbA_1c_, triglycerides, insulin, leptin, hsCRP, and HOMA index, and negatively with HDL cholesterol (Supplemental Table 1). Multiple linear regression identified BMI (*p* > 0.001) and HbA_1c_ (*p* > 0.001) as independent predictors of serum ANGPTL4 (adjusted *R* = 0.552, *p* < 0.001).

ANGPTL3 levels correlated inversely with ANGPTL4 levels (*R* = −0.436, *p* > 0.001). No significant relationship with any of the studied parameters was found for ANGPTL4 mRNA expression.

## Discussion

ANGPTL3 and ANGPTL4 have multiple functions in human body, including a possible role in the regulation of glucose and lipid homeostasis. It is therefore relevant to study changes of their circulating levels and adipose tissue mRNA expression in patients with different nutritional statuses and disturbances in glucose metabolism. In this study we show an opposite association pattern of serum ANGPTL3 and ANGPTL4 with body weight, diabetes status, and parameters of glucose control across a wide range of BMI as well as their different reactions to short- and long-term weight-modifying interventions. Both ANGPTL3 and 4 are potent regulators of triglyceride metabolism influencing triglyceride trafficking and circulating triglyceride levels by selectively inhibiting LPL in key metabolic tissues, including WAT, skeletal muscle, and heart. According to the recently proposed “3-4-8” model based primarily on experimental data ANGPTL4, a selective inhibitor of WAT LPL, is upregulated during fasting shunting thus triglycerides from WAT to skeletal and heart muscle. In contrast, the complex of ANGPTL3 and ANGPTL8 selectively inhibits skeletal and heart LPL, and while ANGPLT3 was reported not to be influenced by nutritional status, ANGPTL8 is upregulated after feeding redirecting the triglyceride flux away from heart and skeletal muscle and into WAT^19^. Here we partially confirm this model, as acute fasting in our subjects almost doubled baseline serum ANGPTL4 levels, which is in line with previous evidence^11,20,21^. Surprisingly, it also significantly decreased serum ANGPTL3, which is in contrast with former data indicating only minimal nutritional regulation of ANGPTL3 synthesis and secretion^11^. However, previous data were derived from in vitro and animal experiments and up to now have not been validated in human subjects. Our results are thus the first to suggest the possibility that ANGPTL3 levels are influenced by acute nutritional status in humans changing in an opposite direction to ANGPTL4. Interestingly, acute refeeding did not affect either ANGPTL levels, most probably owing to the rather short examination period (2 h post refeeding), as in a recent study a significant change in ANGPTL3 and 4 levels could be observed only after 4–6 h postprandially; however, the present trend to reduced postprandial levels corresponds with the published results^22^.

In contrast to acute fasting, chronic malnutrition in subjects with AN had a completely opposite effect on both factors. It markedly decreased serum ANGPTL4 and tended to increase ANGPTL3 as compared with healthy controls. Analogously, the presence of obesity significantly increased ANGPTL4 while tending to reduce ANGPTL3 suggesting body weight as one of the main regulators of ANGPTL levels. This was confirmed by correlation analysis across the whole BMI spectrum with strong positive correlation for ANGPLT4 and BMI and a weaker inverse correlation for ANGPTL3 and BMI, respectively. Interestingly, the presence of T2DM further strengthened the association for ANGPTL4 beyond the sole effect of increased BMI in T2DM compared with OB group, as evidenced by the independent positive association of its levels with HbA_1C_. The influence of T2DM on serum ANGPTL3 was less pronounced; however, significantly decreased ANGPTL3 concentrations in the diabetic group as compared with only an insignificant reduction trend in subjects with simple obesity might indicate a causal interconnection between T2DM and circulating ANGPTL3.

The positive association between ANGPTL4 and BMI potentiated by T2DM in our study confirms previous data^23,24^ as does the much tighter relationship between ANGPTL4 and metabolic parameters compared with ANGPTL3^12^. However, exact mechanisms responsible for these relationships remain largely unidentified. In contrast to the original hypothesis of free fatty acids as main PPAR ligands being the primary regulators of ANGPTL4 synthesis^25^, recent data suggest low-grade inflammation as a more important contributor to increased ANGPTL4 levels in T2DM and the metabolic syndrome^23^. Here we have shown a strong positive correlation between serum ANGPTL4 and hsCRP as a marker of low-grade inflammation that, nevertheless, could not be confirmed in multiple regression analysis. Similarly unclear is the potential role of ANGPTL4 in the development of T2DM and its complications. On one hand, increased ANGPTL4 levels are through inhibition of LPL associated with worsened lipid profile (decreased HDL and increased triglycerides), which was also confirmed in our study^23,26^. On the other hand, overexpression of ANGPTL4 was in experimental studies shown to decrease blood glucose and improve glucose tolerance and insulin resistance^26,27^. The recently proposed model of selective inhibition of LPL in WAT by ANGPTL4 with subsequent rerouting of triglycerides into heart and skeletal muscle might constitute a positive (increased energy influx into tissues) as well as negative mechanism (ectopic accumulation of lipids resulting in increased insulin resistance); to clarify this issue, more in-depth mechanistic studies are required.

Unlike ANGPTL4, the relationship of ANGPTL3 and obesity and T2DM is less clear. In a recent trial in Middle Eastern population, Abu-Farha et al.^24^ showed increased ANGPTL3 levels only in obese non-diabetic subjects as compared to healthy normal-weight controls, whereas in the diabetic group obesity did not affect ANGPTL3 concentrations. The presence of T2DM had no effect on ANGPTL3 regardless of body weight. In contrast, no difference in circulating ANGPTL3 between normal and overweight individuals was observed in a study on pediatric population in Korea^28^. Interestingly, Zhao et al.^29^ reported decreased ANGPTL3 levels in female subjects with T2DM relative to their non-diabetic counterparts (while failing to find any difference in males) as well as increased ANGPTL3 in non-diabetic females compared with males. Similarly, our data show a significant reduction in serum ANGPTL3 in both diabetic groups (VLCD and bariatric surgery) compared to healthy controls, whereas simple obesity was associated only with a nonsignificant trend to reduction. This somehow surprising finding might be partially explained by the substantial prevalence of females in the T2DM subgroup (80%—32 females out of 40 subjects). Another factor contributing to this effect might be severe hyperinsulinemia in diabetic subjects, as high levels of insulin were shown to reduce systemic ANGPTL3 by decreasing its liver expression^30^. These data are of particular interest in the light of recent works showing improved insulin sensitivity after *Angptl3* gene silencing and decreased atherosclerotic development in experimental animals along with improved lipid profile in humans treated with a selective ANGPTL3 inhibitor suggesting thus a possible protective effect of reduced ANGPTL3 levels in T2DM^31–33^.

To date, only limited data on the effect of longer-term weight reduction on serum ANGPTL4 are available, while being virtually non-existent for ANGPTL3. Three days of VLCD (471 kcal/day) increased circulating ANGPTL4 in healthy lean subjects as well as overweight T2DM individuals^25^, while 8 weeks of VLCD (600 kcal/day) raised ANGPTL4 levels by 9% in otherwise healthy obese subjects^34^. Our data show a similar, albeit nonsignificant, trend for ANGPTL4 after 3 weeks of VLCD (600 kcal/day) in both obese and T2DM subjects, while serum ANGPTL3 was conversely reduced in both study groups. Fasting-stimulated elevation of non-esterified fatty acids was suggested as the primary mechanism responsible for the increase in ANGPTL4, while processes influencing ANGPTL3 still need to be elucidated^34^. Interestingly, bariatric surgery-induced weight loss resulted after 1 month in changes similar to VLCD in both ANGPTLs with subsequent reversal into opposite direction after 6 and 12 months suggesting the existence of different regulatory mechanisms of ANGPTL production for (semi-)acute and chronic weight reduction.

SBS as a model of extreme malnutrition was associated with substantially elevated levels of both ANGPTL3 and 4, which might at least partially be attributed to their initially increased proinflammatory status as evidenced by markedly high CRP levels. Interestingly, realimentation had no effect on either ANGPTL as their concentrations remained rather high even after the near-normalization of CRP hinting at the involvement of other regulatory factors than inflammation.

We also evaluated changes of ANGPTL4 mRNA expression in SAT. There were no differences between obese and control subjects or between baseline and post-interventional state, respectively. Moreover, there was no difference between mRNA expression in SAT and VAT samples. These findings together with a lack of significant correlation between mRNA expression in SAT and BMI support the previously published suggestion that adipose tissue has little impact on systemic ANGPTL4 concentrations^35^. Undoubtedly, this suggestion seems to be valid as well for ANGPTL3, which is expressed primarily in liver and kidney. In spite of previously published studies^36,37^, which detected no ANGPTL3 mRNA expression in adipose tissue we found a weak ANGPTL3 mRNA expression in both subcutaneous and VAT confirming thus the findings of Abu-Farha et al.^24^, even though the expression was on the detection limit of the used method and was detectable only in approximately 30% of samples.

In conclusion, we have demonstrated an inverse behavior of ANGPTL3 and 4 across different body weight ranges potentiated by the presence of T2DM, as well as during acute and prolonged weight-reducing interventions. Furthermore, we have shown for the first time the influence of acute fasting on serum ANGPTL3. Taken together, ANGPTL3 and 4 levels are affected by acute as well as chronical nutritional and metabolic status; however, their potential role in the pathogenesis of obesity and T2DM requires further investigation.

## Electronic supplementary material

Supplemental table 1
