# Supplementary material for: Angiopoietin-like protein 3 and 4 in obesity, type 2 diabetes mellitus, and malnutrition: the effect of weight reduction and realimentation
Source: Nutr Diabetes. 2018 Apr 25;8:21. doi: 10.1038/s41387-018-0032-2 (PMC5916880; doi:10.1038/s41387-018-0032-2)
Supplement: Supplementary file 1 — Supplemental table 1 [file 41387_2018_32_MOESM1_ESM.docx]

**Supplemental table 1. Association of ANGPTL3 and ANGPTL4 with other parameters.**

|  | **ANGPTL3** | | **ANGPTL4** | |
| --- | --- | --- | --- | --- |
|  | **R** | **p** | **R** | **p** |
| **Body mass index** | -0.333 | >0.001 | 0.721 | >0.001 |
| **Blood glucose** | -0.276 | >0.001 | 0.603 | >0.001 |
| **HbA_1c_** |  | NS | 0.649 | >0.001 |
| **Triglycerides** | -0.195 | 0.033 | 0.361 | >0.001 |
| **HDL cholesterol** | 0.242 | 0.009 | -0.531 | >0.001 |
| **Insulin** | -0.341 | 0.001 | 0.600 | >0.001 |
| **Leptin** | -0.379 | >0.001 | 0.657 | >0.001 |
| **hsC-reactive protein** |  | NS | 0.596 | >0.001 |
| **HOMA index** | -0.331 | >0.001 | 0.693 | >0.001 |

NS: non-significant result. Spearman or Pearson correlation test.
